# Association of retinal venular tortuosity with impaired renal function in the Northern Ireland Cohort for the Longitudinal Study of Ageing

**DOI:** 10.1186/s12882-020-02031-0

**Published:** 2020-09-03

**Authors:** R. A. O’Neill, A. P. Maxwell, F. Kee, I. Young, B. McGuinness, R. E. Hogg, McKay GJ

**Affiliations:** Centre for Public Health, Queens University Belfast, Belfast, UK

**Keywords:** Retinal microvascular parameters, Renal function, Chronic kidney disease, Estimated glomerular filtration rate

## Abstract

**Background:**

Previous studies have identified retinal microvascular features associated with renal dysfunction. Biopsies are necessary to confirm kidney microvascular damage and retinal imaging may enable evaluation of microangiopathic characteristics reflecting renal changes associated with chronic kidney disease (CKD). We evaluated retinal microvascular parameters (RMPs) for associations with renal function in a cross-sectional analysis of the Northern Ireland Cohort for the Longitudinal Study of Ageing.

**Methods:**

RMPs (central retinal arteriolar/ venular equivalents [CRAE/CRVE], arteriolar to venular ratio [AVR], fractal dimension and tortuosity) were measured from optic disc centred fundus images using semi-automated software. Associations were assessed with multivariable regression analyses between RMPs and estimated glomerular filtration rate (eGFR) defined by serum creatinine (eGFRscr) and cystatin C (eGFRcys) and also CKD status characterised by eGFR < 60 mL/min/1.73m^2^. Regression models were adjusted for potential confounders including age, sex, diabetes, smoking status, educational attainment, cardiovascular disease, body mass index, antihypertensive medication, systolic blood pressure, triglycerides, high- and low-density lipoprotein levels.

**Results:**

Data were included for 1860 participants that had measures of renal function and retinal fundus images of sufficient quality for analysis. Participants had a mean age of 62.0 ± 8.5 yrs. and 53% were female. The mean eGFR for scr and cys were 82.2 ± 14.9 mL/min/1.73m^2^ and 70.7 ± 18.6 mL/min/1.73m^2^ respectively. eGFRcys provided lower estimates than eGFRscr resulting in a greater proportion of participants categorised as having CKD stages 3–5 (eGFRcys 26.8%; eGFRscr 7.9%). Multivariable regression analyses showed that increased venular tortuosity (OR = 1.30; 95%CI: 1.10, 1.54; *P* < 0.01) was associated with CKD stages 3–5 characterised by eGFRscr < 60 mL/min/1.73 m^2^. No additional associations between CKD status characterised by eGFRscr or with eGFRcys, were detected (*P* > 0.05). Multivariable regression failed to detect associations between CRAE, CRVE, AVR, fractal dimension or tortuosity and eGFRscr or eGFRcys (*P* > 0.05).

**Conclusion:**

Increased retinal venular tortuosity was associated with CKD stages 3–5 defined by eGFRscr < 60 mL/min/1.73 m^2^, in an older population independent of potential confounding factors. These retinal measures may provide non-invasive microvascular assessment of associations with CKD.

## Background

Global increases in ageing have been widely reported and those > 50 years (yrs) represent the most rapidly expanding demographic in the Northern Ireland population [1]. Social economic development and heath care improvements have increased life expectancy impacting upon societal health care systems, policies and demands [2–4]. Chronic kidney disease (CKD) is a major global health concern with estimates suggesting between 3 and 18% of the population are affected, [1, 2] leading to substantial economic burden [3, 4] and diminished quality of life [5]. CKD incidence and prevalence is greatest among the elderly [3] and is expected to increase further over the coming decades as populations age [6]. CKD is characterised by irreversible reductions in the excretory and homeostatic functions of the kidneys [7] leading to a higher risk of adverse outcomes including cardiovascular mortality [8], and is predicted to become the fifth most common cause of death worldwide by 2040 [9]. Improved non-invasive, early-stage kidney disease detection would offer clinical utility for the identification of individuals at increased risk of CKD for targeted intervention to limit the extent and rate of kidney function loss [10].

Common biomarkers of renal function and damage include serum creatinine, cystatin C, and proteinuria but their ability to identify those at greatest risk of future decline is limited [1]. Several biomarkers have improved CKD detection and risk prediction [11] and while tissue-derived markers have proven useful for the identification of accumulated renal microvascular damage [12–16], they tend to be less amenable to non-invasive assessment [17].

Microvascular pathology is commonly found in eye and kidney diseases with several studies reporting associations between renal impairment and retinal microvascular variation although the findings have not always been consistent [12–16]. Such associations may be indicative of systemic vascular effects and renovascular damage [18]. Furthermore, similarities in the cellular physiological characteristics that characterise the renal and retinal microcirculation, including retinal pericytes and renal mesangial cells, implicate similar pathological pathways leading to end organ damage [19]. Advances in retinal imaging modalities and analysis applications readily identify microangiopathic variation in the eye. These innovations provide novel opportunities to assess if specific retinal anatomical features can be correlated with measures of kidney function [17, 20, 21]. As such, the aim of this study was to assess retinal microvascular parameters (RMPs) in association with baseline measures of renal function in a cross-sectional analysis of older persons from the Northern Ireland Cohort for the Longitudinal Study of Ageing (NICOLA).

## Methods

### Study characteristics

NICOLA is a longitudinal cohort study of 8468 community dwelling men and women aged 50 years and over, resident in Northern Ireland (individuals in care homes or other residential institutions were excluded at baseline) [22]. The study, established in 2012, has three main components: a computer aided personal interview (CAPI), a self-completion questionnaire and health assessment (Supplementary file 1). The CAPI was extensive in scope and included assessment of demographic, social and health-related factors, and was conducted at individual appointments in each participant’s home between December 2013 and March 2016. Measures of cardiovascular, physical, cognitive and visual function were determined and a biobank of biological samples collected simultaneously which included visual health with retinal fundus photography. Written informed consent was obtained from participants prior to participation under ethical approval from the School of Medicine, Dentistry and Biomedical Sciences Ethics Committee, Queen’s University Belfast (SREC 12/23) and in accordance with the Helsinki Declaration.

### Measurement of renal function and classification of CKD

Serum creatinine (scr, mg/dL) standardised to isotope dilution mass spectrometry (IDMS) calibrated techniques and cystatin C (cys, mg/L) were assayed on an Abbott ARCHITECT c8000 system using kinetic alkaline picrate and turbidimetric/ immunoturbidimetric methods, respectively. The coefficient of variation for creatinine and cystatin C was < 4.68 and < 1.80% respectively. Estimated glomerular filtration rate (eGFRscr and eGFRcys) was based on a single serum sample using the Chronic Kidney Disease Epidemiology Collaboration equation (CKD-EPI 2009 equation for scr and 2012 equation for cys) [23, 24]. CKD stages 3–5 were defined as eGFR < 60 mL/min/1.73m^2^ and CKD stages 1–2 as eGFR ≥60 mL/min/1.73m^2^.

### Other variables

**Fig. 1:**
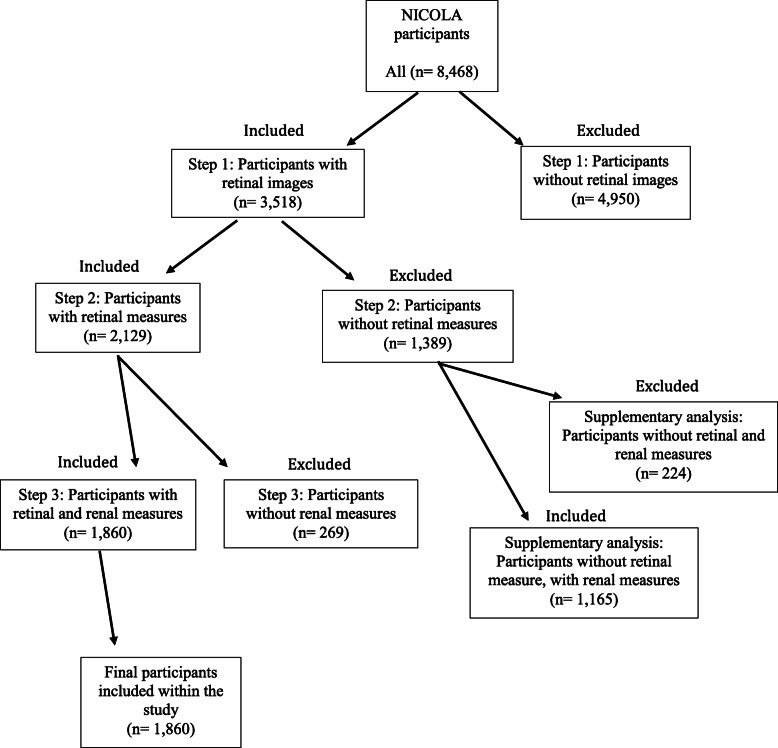
A flow chart of participant inclusion and exclusion criteria

Systolic blood pressure (SBP) was calculated as the average of two individual measurements. Diabetes status was characterised as a combination of HbA1c ≥ 6.5%, use of diabetic medications and self-reported diabetes at CAPI and health assessment. Educational attainment was dichotomised: primary and lower or secondary level and above (including university education). Smoking status was categorised as current smokers versus non-smokers. Cardiovascular disease (CVD) was by self-report and characterised by a history of angina, heart attack, congenital heart failure or stroke. Participants were excluded if scr or cys were missing or retinal images were of insufficient quality for image analysis (Fig. 1).

### Retinal image acquisition and measurement

**Fig. 2:**
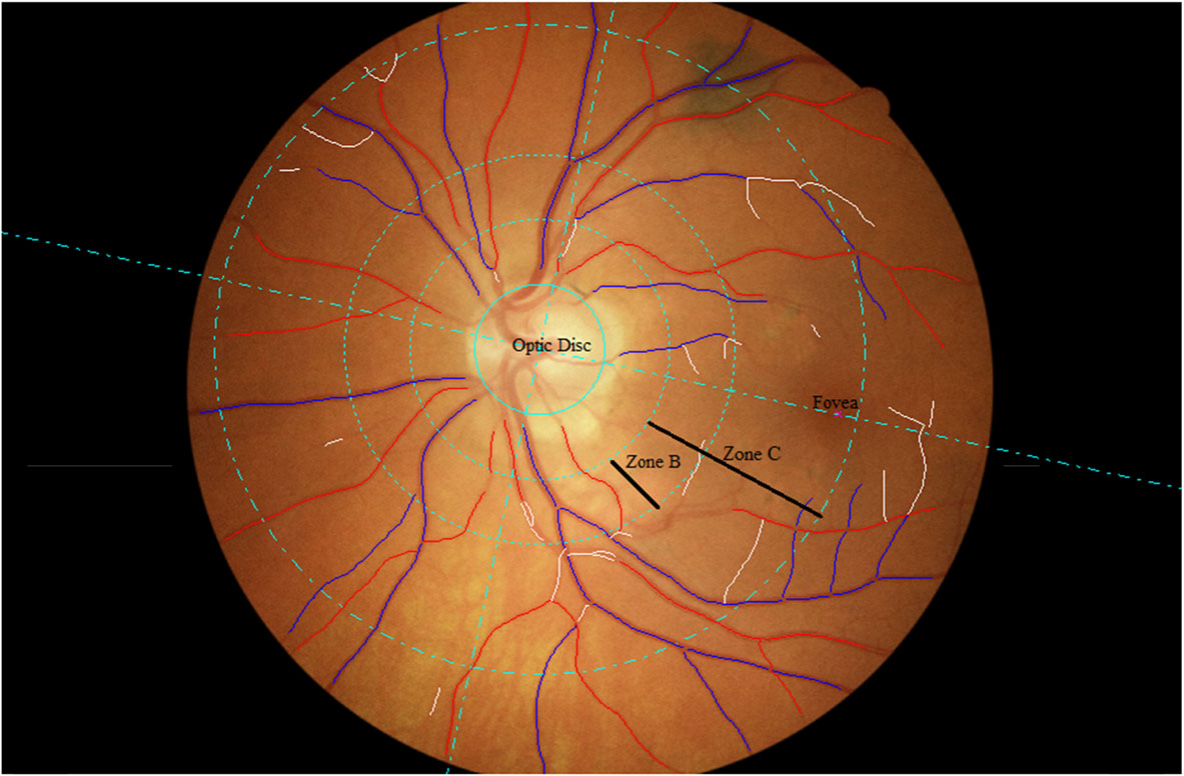
Optic disc centred retinal fundus image assessed using the Vessel Assessment and Measurement Platform for Images of the Retina (VAMPIRE) software. Arterioles (red), venules (blue) and deleted segments (white) are indicated. The retinal microvascular parameters for arteriolar and venular calibre (CRAE, CRVE, and AVR), fractal dimension and tortuosity are calculated from measurements captured in zones B and C (1.0 to 2.5 optic disc diameters from the disc margin)

Retinal photography was performed through the dilated pupil using a Canon CX-1 Digital Fundus Camera (Canon USA, Melville, NY, USA), following dilation from a single drop of 1% tropicamide in all participants. RMPs (central retinal arteriolar/ venular equivalents [CRAE/CRVE], arteriolar to venular ratio [AVR], fractal dimension and tortuosity) were measured from optic disc centred fundus images, collected at participant health assessment and analysed using the semi-automated software Vessel Assessment and Measurement Platform for Images of the Retina (VAMPIRE; VAMPIRE group, University of Dundee, Dundee, Scotland, Version 3.1; Fig. 2), by a qualified grader blinded to participant data [25, 26]. Images were taken from the left eye except when unavailable or of poor quality, in which case the right eye image was used. Intraclass correlation coefficients (ICCs) were calculated to assess intergrader reliability with mean values of 0.87 (CRAE) and 0.91 (CRVE).

### Statistical analysis

All analyses were performed using Statistical Package for Social Sciences (Version 24.0. Armonk, NY: IBM Corp). Before inclusion in regression models all RMPs were transformed into standardised Z-scores. Independent samples t-tests and chi-squared tests were used to compare the distribution of demographic factors and clinical variables between participants characterised as CKD stages 1–2 and CKD stages 3–5. Population characteristics were described using mean and standard deviation (SD) for continuous variables or frequencies and percentages for categorical variables. Linear and logistic regression were used to evaluate associations between RMPs and renal function (eGFRscr and eGFRcys) and the binary trait of CKD status. Minimally adjusted models included age and sex with fully adjusted models also including diabetes and smoking status, educational attainment, CVD, body mass index (BMI), antihypertensive medication, systolic blood pressure, triglycerides, high and low-density lipoprotein (HDL and LDL) levels. *P* < 0.05 was considered statistically significant.

## Results

From 8468 participants, data was available for 1860 participants with measures of renal function and retinal fundus images of sufficient quality (Fig. 1). Participant characteristics are summarised in Table 1; comparisons between participants with retinal fundus images included with in the study or not, are summarised in Supplementary Table 1. A STROBE Statement (STrengthening the Reporting of OBservational studies in Epidemiology) is a reporting guideline that includes a checklist of 22 items considered essential for good reporting of observational studies and is presented in Supplementary Table 2. Participants had a mean age of 62.0 ± 8.5 yrs. and 53% were female. The mean eGFR for scr and cys were 82.2 ± 14.9 mL/min/1.73m^2^ and 70.7 ± 18.6 mL/min/1.73m^2^ respectively. SBP was 131 ± 18.5 mmHg and 23% of participants had diabetes mellitus. There were 147 participants with CKD stages 3–5 characterised by eGFRscr < 60 mL/min/1.73 m^2^ compared to 498 using eGFRcys (CKD stages 3–5 = 7.9% vs 26.8% respectively). As expected, participants with CKD stages 3–5 had significantly lower mean eGFR compared to participants with CKD stages 1–2 (eGFRscr: 49.6 ± 7.8 mL/min/1.73 m^2^ versus 85.0 ± 11.7 mL/min/1.73 m^2^, and eGFRcys: 49.1 ± 8.0 mL/min/1.73 m^2^ versus 78.6 ± 14.7 mL/min/1.73 m^2^). Participants with CKD stages 3–5 had a significantly higher mean age compared to participants with CKD stages 1–2 (eGFRscr: 70.5 ± 9.0 yrs. versus 61.3 ± 8.1 yrs. and eGFRcys: 68.2 ± 8.3 yrs. versus 59.8 ± 7.4 yrs). The difference between eGFRscr and eGFRcys estimates against mean eGFR, is presented in a Bland Altman plot (Supplementary Figure 1). A lower percentage of participants in CKD stages 3–5 were female than CKD stages 1–2 (eGFRscr: 44% versus 54% and eGFRcys: 51% versus 54%). Higher percentages of individuals in CKD stages 3–5 for both eGFRscr and eGFRcys were taking antihypertensive medication (eGFRscr: 46% versus 25% and eGFRcys: 42% versus 22%) and had a history of CVD (eGFRscr: 22% versus 7% and eGFRcys: 14% versus 5%; Table 1).

**Table 1:** Participant summary characteristics comparisons for CKD stages 1–2 and CKD stages 3–5

**Participant characteristic scr**	**All (*n*** **= 1860)**	**CKD stages 1–2** **(*n*** **= 1713)**	**CKD stages 3–5** **(*n*** **= 147)**	***P*** **Value**
Mean age (years, SD)	62.0 ± 8.5	61.3 ± 8.1	70.5 ± 9.0	< 0.01
Female, n (%)	994 (53.4)	930 (54.3)	64 (43.5)	0.01
Education, primary level and below, n (%)	238 (12.8)	210 (12.6)	28 (19.0)	0.02
Diabetes, yes n (%)	429 (23.1)	380 (22.2)	49 (33.3)	< 0.01
Mean BMI (kg/m^2^, SD)	28.6 ± 5.0	28.5 ± 5.0	29.2 ± 4.4	0.12
Mean systolic blood pressure (mmHg, SD)	131.1 ± 18	131 ± 18	133 ± 19	0.27
Antihypertension medication, yes n (%)	506 (27.2)	438 (25.6)	68 (46.3)	< 0.01
Mean triglyceride (mmol/L, SD)	1.7 ± 1.0	1.6 ± 1.0	1.8 ± 0.8	0.11
Mean HDL cholesterol (mmol/L, SD)	1.6 ± 0.5	1.6 ± 0.4	1.5 ± 0.5	< 0.01
Mean LDL cholesterol (mmol/L, SD)	3.5 ± 1.1	3.5 ± 1.1	3.1 ± 1.2	< 0.01
Cardiovascular disease, yes n (%)	143 (7.7)	111 (6.5)	32 (21.8)	< 0.01
Mean eGFR Creatinine (mL/min/1.73m^2^, SD)	82.2 ± 14.9	85.0 ± 11.7	49.6 ± 7.8	< 0.01
**Participant characteristic cys**	**All (*n*** **= 1860)**	**CKD stages 1–2 (*n*** **= 1362)**	**CKD stages 3–5 (*n*** **= 498)**	***P*** **Value**
Mean age (years, SD)	62.0 ± 8.5	59.8 ± 7.4	68.2 ± 8.3	< 0.01
Female, n (%)	994 (53.4)	739 (54.3)	255 (51.2)	0.24
Education, primary level and below, n (%)	238 (12.8)	138 (10.1)	100 (20.1)	< 0.01
Diabetes, yes n (%)	429 (23.1)	271 (19.9)	158 (31.7)	< 0.01
Mean BMI (kg/m^2^, SD)	28.6 ± 5.0	28.0 ± 4.5	30.3 ± 5.6	< 0.01
Mean systolic blood pressure (mmHg, SD)	131.1 ± 18	130 ± 19	133 ± 18	< 0.01
Antihypertension medication, yes n (%)	506 (27.2)	297 (21.8)	209 (42.0)	< 0.01
Mean triglyceride (mmol/L, SD)	1.7 ± 1.0	1.6 ± 0.9	1.8 ± 1.0	< 0.01
Mean HDL cholesterol (mmol/L, SD)	1.6 ± 0.5	1.7 ± 0.5	1.6 ± 0.4	< 0.01
Mean LDL cholesterol (mmol/L, SD)	3.5 ± 1.1	3.5 ± 1.1	3.2 ± 1.2	< 0.01
Cardiovascular disease, yes n (%)	143 (7.7)	73 (5.4)	70 (14.1)	< 0.01
Mean eGFR Cystatin C (mL/min/1.73m^2^, SD)	70.7 ± 18.6	78.6 ± 14.7	49.1 ± 8.0	< 0.01

Values are n (%) for categorical variables and mean ± SD for continuous variables. *P* values were calculated by independent sample t and chi squared tests. *Abbreviations*: *CKD* Chronic Kidney Disease, *BMI* Body Mass Index, *eGFR* Estimated glomerular filtration rate (calculated using the CKD-EPI equation), *HDL* High-density Lipoprotein, *LDL* Low-density Lipoprotein, *scr* Serum Creatinine, *cys* Serum Cystatin C, *SD* Standard deviation

Venular tortuosity was associated with an increased risk of CKD stages 3–5 characterised by eGFRscr in all models (fully adjusted odds ratio [OR] =1.30; 95% CI: 1.10, 1.54; *P* < 0.01, respectively), although this was not the case for CKD stages 3–5 defined by eGFRcys (*P* > 0.05; Table 2). Venular fractal dimension was associated with a reduced risk of CKD stages 3–5 characterised by eGFRscr (OR = 0.84; 95% CI: 0.70, 1.00; *P* = 0.05) but did not survive adjustment for the potential confounders age, sex, diabetes and smoking status, educational attainment, BMI, antihypertensive medication, CVD, SBP, triglycerides, HDL and LDL (OR = 0.87; 95% CI: 0.72, 1.05; *P* = 0.15). Arteriolar fractal dimension (OR = 0.90; 95% CI: 0.82, 1.00; *P* = 0.05) and CRVE (OR = 1.13; 95% CI: 1.02, 1.25; *P* = 0.02) were associated with risk of CKD stages 3–5 characterised by eGFRcys but again, did not survive adjustment for confounding factors. No further associations were detected between other RMPs and CKD stages 3–5 (*P* > 0.05; Table 2).

**Table 2:** Association of retinal vessel parameters and CKD status using binary logistic regression (scr and cys)

**CKD status scr**	**Unadjusted**			**Minimally adjusted**			**Fully adjusted**		
**Retinal parameter**	**OR**	**95% CI**	***P*** **Value**	**OR**	**95% CI**	***P*** **Value**	**OR**	**95% CI**	***P*** **Value**
^a^CRAE (PX)	1.17	1.00,1.38	0.06	1.12	0.94, 1.34	0.20	1.15	0.95, 1.38	0.14
^a^CRVE (PX)	1.10	0.93, 1.30	0.27	1.06	0.89, 1.26	0.54	1.03	0.86, 1.23	0.76
^a^AVR	1.06	0.89, 1.25	0.53	1.04	0.87, 1.25	0.64	1.09	0.91, 1.30	0.37
^a^Fractal dimension arteriolar	1.02	0.86, 1.21	0.83	1.10	0.91, 1.32	0.33	1.09	0.90, 1.32	0.36
^a^Fractal dimension venular	0.86	0.73, 1.00	0.05	0.89	0.75, 1.07	0.22	0.87	0.72, 1.05	0.15
^ab^Tortuosity arteriolar	1.14	0.96, 1.34	0.14	1.13	0.94, 1.35	0.19	1.10	0.91, 1.32	0.33
^ab^Tortuosity venular	1.36	1.16, 1.59	< 0.01	1.34	1.14, 1.59	< 0.01	1.30	1.10, 1.54	< 0.01
**CKD status cys**	**Unadjusted**			**Minimally adjusted**			**Fully adjusted**		
**Retinal parameter**	**OR**	**95% CI**	***P*** **Value**	**OR**	**95% CI**	***P*** **Value**	**OR**	**95% CI**	***P*** **Value**
^a^CRAE (PX)	1.09	0.98, 1.21	0.10	1.05	0.94, 1.18	0.39	1.09	0.96, 1.23	0.19
^a^CRVE (PX)	1.13	1.02, 1.25	0.02	1.12	1.00, 1.25	0.06	1.08	0.96, 1.22	0.21
^a^AVR	0.97	0.87, 1.07	0.55	0.94	0.84, 1.06	0.33	1.00	0.89, 1.14	0.97
^a^Fractal dimension arteriolar	0.90	0.82, 1.00	0.05	0.95	0.84, 1.06	0.33	0.98	0.87, 1.10	0.69
^a^Fractal dimension venular	0.92	0.83, 1.02	0.10	0.98	0.87, 1.10	0.68	0.98	0.87, 1.11	0.77
^ab^Tortuosity arteriolar	1.07	0.96, 1.18	0.23	1.06	0.94, 1.18	0.37	1.03	0.91, 1.16	0.66
^ab^Tortuosity venular	1.10	1.00, 1.22	0.06	1.08	0.97, 1.21	0.17	1.03	0.92, 1.17	0.59

*Abbreviations*: *eGFR* Estimated glomerular filtration rate (Calculated using the CKD-EPI equation), *CKD* Chronic Kidney Disease, *CRAE* Central Retinal Arteriolar Equivalent, *CRVE* Central Retinal Venular Equivalent, *AVR* Retinal Arteriole/Venular Ratio, *scr* Serum Creatinine, *cys* Serum Cystatin C, *CI* Confidence Interval, *OR* Odds Ratio, *PX* Pixels. ^a^RMPs were transformed into standardised Z-scores before inclusion in regression models. ^b^Tortuosity values were log transformed before inclusion in regression models to produce normal distribution. Minimally adjusted models included age (yrs) and sex, with fully adjusted models also including diabetes and smoking status, cardiovascular disease, educational attainment, body mass index, antihypertensive medication, systolic blood pressure, triglycerides, high and low-density lipoproteins levels. *P* values and 95% confidence intervals were generated from the regression models

In an unadjusted linear regression, CRAE (β [Beta] = − 0.83; 95% CI: − 1.50, − 0.15; *P* = 0.02) and venular fractal dimension (β = 0.81; 95% CI: 0.14, 1.49; *P* = 0.02) were associated with eGFRscr but did not survive adjustment for potential confounders in minimally or fully adjusted models (*P* > 0.05; Table 3). CRVE was associated with eGFRcys in an unadjusted linear regression model (β = − 1.23; 95% CI: − 2.08, − 0.38; *P* = < 0.01) but associations did not survive in the minimally adjusted or fully adjusted models. No further associations were detected between RMPs assessed as standardised Z-scores in a linear regression analysis with eGFRscr and eGFRcys (*P* > 0.05; Table 3).

**Table 3:** Association of retinal vessel parameters and eGFR using linear regression (scr and cys)

**eGFR scr**	**Unadjusted**			**Minimally adjusted**			**Fully adjusted**		
**Retinal parameter**	**β**	**95% CI**	***P*** **Value**	**β**	**95% CI**	***P*** **Value**	**β**	**95% CI**	***P*** **Value**
^a^CRAE (PX)	−0.83	−1.50, −0.15	0.02	−0.47	− 1.06, −.12	0.12	− 0.54	− 1.13, 0.06	0.08
^a^CRVE (PX)	−0.17	− 0.84, 0.51	0.63	0.11	−0.48, 0.70	0.72	0.11	− 0.48, 0.69	0.72
^a^AVR	−0.56	−1.24, 0.12	0.11	− 0.49	− 1.08, 0.10	0.11	− 0.54	− 1.13, 0.05	0.07
^a^Fractal dimension arteriolar	0.27	−0.41, 0.94	0.44	−0.14	−0.73, 0.46	0.65	−0.03	− 0.62, 0.56	0.92
^a^Fractal dimension venular	0.81	0.14, 1.49	0.02	0.35	−0.24, 0.94	0.25	0.41	−0.18, 1.00	0.17
^ab^Tortuosity arteriolar	−0.37	−1.05, 0.31	0.28	−0.23	−0.82, 0.36	0.44	−0.18	− 0.78, 0.40	0.53
^ab^Tortuosity venular	−0.47	−1.14, 0.21	0.18	−0.27	−0.87, 0.32	0.36	−0.16	− 0.74, 0.43	0.59
**eGFR cys**	**Unadjusted**			**Minimally adjusted**			**Fully adjusted**		
**Retinal parameter**	**β**	**95% CI**	***P*** **Value**	**β**	**95% CI**	***P*** **Value**	**β**	**95% CI**	***P*** **Value**
^a^CRAE (PX)	−0.68	−1.53, 0.17	0.12	−0.34	−1.07, 0.40	0.37	−0.46	−1.17, 0.26	0.21
^a^CRVE (PX)	−1.23	−2.08, −0.38	< 0.01	−0.87	− 1.60, − 0.13	0.02	− 0.53	− 1.23, 0.17	0.14
^a^AVR	0.55	− 0.30, 1.39	0.21	0.54	−0.20, 1.28	0.15	0.12	−0.59, 0.83	0.73
^a^Fractal dimension arteriolar	0.55	−0.29, 1.40	0.20	−0.04	−0.78, 0.70	0.92	−0.15	− 0.85, 0.55	0.68
^a^Fractal dimension venular	0.14	−0.71, 0.99	0.75	−0.48	−1.22, 0.26	0.20	−0.33	− 1.03, 0.37	0.35
^ab^Tortuosity arteriolar	−0.69	−1.53, 0.16	0.11	−0.56	−1.29, 0.18	0.14	−0.43	−1.13, 0.27	0.23
^ab^Tortuosity venular	−0.82	−1.67, 0.03	0.06	−0.53	−1.26, 0.21	0.16	−0.24	−0.94, 0.45	0.49

*Abbreviations*: *eGFR* Estimated glomerular filtration rate (Calculated using the CKD-EPI equation), *CRAE* Central Retinal Arteriolar Equivalent, *CRVE* Central Retinal Venular Equivalent, *AVR* Retinal Arteriole/Venular Ratio, *scr* Serum Creatinine, *cys* Serum Cystatin C, *CI* Confidence Interval, *β* Beta, *PX* Pixels. ^a^RMPs were transformed into standardised Z-scores before inclusion in regression models. ^b^Tortuosity values were log transformed before inclusion in regression models to produce normal distribution. Minimally adjusted models included age (yrs) and sex, with fully adjusted models also including, diabetes and smoking status, cardiovascular disease, educational attainment, body mass index, antihypertensive medication, systolic blood pressure, triglycerides, high and low-density lipoproteins levels. P values and 95% confidence intervals were generated from the regression models

## Discussion

The eye provides an opportunistic non-invasive evaluation of the retinal microvasculature that may represent ongoing microvascular pathology elsewhere in the body. The majority of previously published studies have used measures of retinal vascular calibre (CRAE, CRVE and AVR), with only a small number evaluating other RMPs such as fractal dimension and tortuosity [27, 28]. Benitez-Aguirre and colleagues reported associations between venular tortuosity and incident renal dysfunction in a prospective cohort of 511 adolescents with type 1 diabetes [29]. Our findings also identify increased venular tortuosity in association with CKD status characterised by eGFRscr < 60 mL/min/1.73m^2^. Notably, the association was independent of a broad array of potential confounders including age, sex, diabetes and smoking status, educational attainment, CVD, BMI, antihypertensive medication, SBP, triglycerides, HDL and LDL levels (OR = 1.30; 95% CI: 1.10, 1.54; *P* < 0.01). The number of individuals characterised with CKD stages 3–5 based on eGFRscr, was considerably lower in contrast to those characterised by eGFRcys (147 versus 498, representing 8% or 27% of the study participants respectively, Table 1). The mechanisms that underlie venular tortuosity are unclear. Low levels of vessel tortuosity are not uncommon in the absence of overt clinical symptoms and high levels of vessel tortuosity have been reported in association with ischaemic changes in more distal organs [30]. Tortuous retinal vessels have been reported as the first identifiable vascular change in many retinopathies and in association with vascular disease, older age, lower HDL levels, hypertension, diabetes and genetic disorders such as hereditary vascular retinopathy and familial retinal arteriolar tortuosity [27–37]. Vessel tortuosity may result from mechanical instability under the influence of haemodynamic alterations that include endothelial dysfunction and blood flow leading to vessel remodelling [29]. Interestingly, the association between venular tortuosity and CKD stages 3–5 was strongest among the 147 participants with poorer renal function for both estimating equations compared to the 351 additional participants identified using the cystatin C equation only (mean eGFRscr: 49.6 [*n* = 147] versus 75.4 [*n* = 351] mL/min/1.73m^2^ and eGFRcys: 45.1 [*n* = 147] versus 51.5 [*n* = 351] mL/min/1.73m^2^; data not shown). As such, retinal microvascular tortuosity may reflect changes in the kidney associated with CKD stages 3–5 but given the cross-sectional nature of this study, it is not possible to differentiate cause and effect [30, 38].

Several cross-sectional studies have previously reported associations between RMPs and renal dysfunction [16, 18, 39, 40] including arteriolar narrowing in those with eGFR < 60 mL/min/1.73m^2^ [41, 42]. In contrast, other studies have failed to detect associations between RMPs and renal function [43, 44]. We failed to detect any associations between CRAE and CKD stages 3–5 defined as eGFR < 60 mL/min/1.73m^2^. A study by Edwards and colleagues hypothesised that abnormalities in the retinal microvasculature were associated with renal dysfunction in an elderly population. However, despite reported associations between retinopathy and renal function, no associations between retinal arteriolar or venular calibre and renal function were detected [45]. Similarly, our study also failed to detect any association between RMPs and the continuous measure of eGFR (scr or cys) or the dichotomised variable of CKD status characterised by eGFRcys.

Our study had several limitations. Firstly, although not routinely measured in population-based studies, the absence of ACR limited the characterisation of renal function in this older population, which was reliant on single measures of scr and cys. Although single serum measures of scr or cys is common in population-based epidemiological studies it differs from clinical CKD staging which, in the absence of proteinuria, depends on two measures of eGFR < 60 mL/min/1.73m^2^ at least 3 months apart [46]. Single eGFR measures sampled from population-based studies usually represent a reliable estimate in comparison to clinically confirmed cases, which can show greater variability. As such, population-based studies may not accurately reflect clinically observed eGFR and may not be directly comparable to clinically confirmed CKD. Secondly, the predominantly white study population aged greater than 50 yrs. may limit the generalisability of our findings to other populations. Thirdly, although NICOLA is a longitudinal study, data was only available for baseline measures facilitating analyses of cross-sectional associations between RMPs and renal function which are not indicative of the potential predictive capacity any associations may represent. Finally, although we adjusted for major potential confounders, the possibility of residual confounding by variables that were not included in the analyses remains.

Despite these limitations, our study had several strengths including the population-based design. NICOLA provided a robust study size with well-characterised individuals with a wide range of demographic factors and clinical variables including co-morbidities and medications, enabling adjustment for potential confounding factors. In addition, the availability of optic disc centred retinal fundus images provides a more robust determination of RMPs using in silico tools in comparison to studies reliant on macula centred images, which are largely confined to evaluation of the microvasculature in the retinal temporal arcades. Furthermore, we used the CKD-EPI equation which is considered a more reliable measure of renal function, particularly at higher eGFR values [47]. Previous studies have largely defined eGFR based on scr to characterise renal function. We used both scr and cys to estimate GFR and characterise CKD status. Previous studies have reported variation in the characterisation of renal function when using eGFRcys or eGFRscr CKD classification [24, 48–50]. Husain and colleagues compared eGFRcys (CKD-EPI 2012 equation) and eGFRscr (CKD-EPI 2009 equation) to classify CKD status in an elderly cohort. They reported a lower mean eGFRcys of 23 ± 15 mL/min/1.73m^2^ compared to eGFRscr, leading to a higher classification of CKD prevalence (71% vs 22% respectively, *P* = < 0.001; 49). On average, the mean eGFRcys for all participants in the current study was 11.5 mL/min/1.73m^2^ lower than the mean eGFRscr, and 0.5 mL/min/1.73m^2^ lower for those with CKD stages 3–5 and 6.4 mL/min/1.73m^2^ lower for those classified with CKD stages 1–2, representing a mean greater difference in those with better renal function. Several advantages have been proposed for the use of cystatin C over serum creatinine as a more sensitive estimate of renal function. Cystatin C is released by all cell types, freely filtered by the kidney and is less confounded by diet, ethnicity, muscle mass or sex [51]. Lees and colleagues (2019) demonstrated improved sensitivity of cystatin C-based eGFR for the prediction of all-cause mortality and fatal/nonfatal CVD in 440,526 participants of the UK Biobank study [51]. As such, we provide data that examines associations using both eGFR determinants of renal function for comparative purposes.

Retinal imaging offers a novel opportunity to complement CKD screening in different clinical and public health settings. Individuals with CKD typically remain asymptomatic for a prolonged period (years) and appropriate screening would facilitate targeted implementation of preventive measures to reduce health-care burden. Moreover, CKD awareness is as low as 10% and studies to identify the potential benefits and risks of screening, screening measures, and target groups for screening of asymptomatic individuals, would help inform inconsistent screening guidelines that exist across professional bodies [52]. Advances in retinal fundus imaging technology and integration with machine learning approaches will enable rapid, non-invasive, point-of-care diagnoses, which may enhance screening service provision and improved screening compliance [53]. Retinal cameras are common in primary care settings and high street opticians for diabetic retinopathy screening. Recent advances in smartphone technology, combined with the utility of machine learning approaches, highlights the feasibility and potential offered by non-invasive retinal photography as an adjunctive or opportunistic screening tool for CKD in the community [53].

## Conclusions

In summary, our findings identify increased variation in retinal venular tortuosity in association with poorer renal function (specifically CKD status characterised by scr) in an older population. These non-invasive retinal measures may help identify mechanistic pathways of microvascular variation early in the disease process in individuals at increased risk of CKD stages 3–5. Identification of such individuals may offer clinical utility for the stratification of those most in need of more frequent surveillance and earlier therapeutic intervention to limit the extent of disease progression.

## Supplementary information

**Additional file 1: Supplementary Table 1**. Comparison of demographic characteristics between all participants with retinal fundus imaging with and without VAMPIRE retinal measures. **Supplementary Table 2**. STROBE Statement—Checklist of items that should be included in reports of cross-sectional studies. **Supplementary Figure 1**.

**Additional file 2:.** Supplementary file 1.

## Data Availability

The data that support the findings of this study are available from the Northern Ireland Cohort of Longitudinal Ageing but restrictions apply to the availability of this data, which was used under license for the current study, and so is not publicly available. Data may however available from the corresponding authors upon reasonable request and provided there is permission from NICOLA.

## Abbreviations

NICOLA: The Northern Ireland Cohort for Longitudinal Ageing
CKD: Chronic kidney disease
CKD-EPI: Chronic Kidney Disease Epidemiology Collaboration eq.
BMI: Body Mass Index
eGFR: Estimated glomerular filtration rate
scr: Serum Creatinine
cys: Serum Cystatin C
HDL: High-density Lipoprotein
LDL: Low-density Lipoprotein
CRAE: Central Retinal Arteriolar Equivalent
CRVE: Central Retinal Venular Equivalent
AVR: Retinal Arteriole/Venular Ratio
SD: Standard deviation
PX: Pixels
eGFRscr: Estimated glomerular filtration rate from serum creatinine
eGFRcys: Estimated glomerular filtration rate from serum cystatin C
NI: Northern Ireland
CPH: Centre for Public Health
SBP: Systolic blood pressure
DBP: Diastolic blood pressure
CI: Confidence intervals
CKD scr: Chronic kidney disease classification from serum creatinine
CKD cys: Chronic kidney disease classification from serum cystatin C
β: Beta value
μm: Microns
ICC’s: Intraclass correlation coefficients
VAMPIRE: Vessel Assessment and Measurement Platform for Images of the Retina
MABP: Mean arteriole blood pressure
RMPs: Retinal microvascular parameters
Yrs: Years
ACR: Urine albumin to creatinine ratio
CVD: Cardiovascular disease
SBP: Systolic blood pressure
