# Supplementary material for: Association of retinal venular tortuosity with impaired renal function in the Northern Ireland Cohort for the Longitudinal Study of Ageing
Source: BMC Nephrol. 2020 Sep 3;21:382. doi: 10.1186/s12882-020-02031-0 (PMC7469276; doi:10.1186/s12882-020-02031-0)
Supplement: Supplementary file 2 — Additional file 2:. Supplementary file 1. [file 12882_2020_2031_MOESM2_ESM.pdf]

NICOLA STUDY **Version 1.9 270515** **Wave 1**

NICOLA ID: | | | | | | |

Exclusion Criteria

Has the participant:

Question		If Yes Answered Exclude from :
Had recent eye surgery or detached retina in the past 4 weeks?	**Yes / No**	Spirometry/ IOP/ Pupil dilation, AF images Optos & Spectralis
Had retinal/ subconjunctival haemorrage in past 3 months	**Yes / No**	Spirometry
Photo-sensitive epilepsy?	**Yes / No**	Pupil dilation/ Canon/ Optus/Spectralis
Ever had a reaction to Tropicamide eye drops?	**Yes / No**	Tropicamide eye drops, AF images Optos & Spectralis
Had hand/wrist surgery in past 6 months?	**Yes / No**	Grip strength
Hand/wrist: swelling, inflammation, severe pain, recent injury?	**Yes / No**	Grip strength
A pace-maker or electrical implantable device?	**Yes / No**	Bodystat
Possibility of Pregnancy?	**Yes / No**	Bodystat
Currently on medication for TB?	**Yes / No**	Spirometry
Had a chest infection/severe cold in the past 4 weeks?	**Yes / No**	Spirometry
Had a heart attack in the past 3 months?	**Yes / No**	Spirometry
History of cerebral, abdominal or aortic aneuryism?	**Yes / No**	Spirometry
Had a pneumothorax in the past 1 year?	**Yes / No**	Spirometry
Had a pulmonary embolism in past 3 months	**Yes / No**	Spirometry
Had any abdominal or chest surgery in last 3 months	**Yes / No**	Spirometry

**Pre Health Assessment check list**		
Are you Diabetic	**Yes / No**	
Any Allergies	**Yes / No**	**If Yes Record Here:**
**Pre discharge check list**		
Warwick Edinburgh Questionaire	**Yes / No**	
Travel Expenses Form	**Yes / No**	
Nutrition Self Completion Booklet	**Yes / No**	
Self Completion Questionaire Returned	**Yes / No**	
Copy Consent Form	**Yes / No**	
**COMMENTS:**		
**Recorded By:**	**Signature:**	**Date:** _ _ / _ _ _ / _ _ _ _

COMMENTS			
1	Refused Test	4	Test Incomplete
2	Physically Unable	5	Test Abandoned
3	Best Attempt	6	Other (please State)

NICOLA ID: | | | | | | |

**Age**	_ _ **Years**	**Gender**	**M/F**	**Comments** (Insert Code)
**Start Time**	_ _:_ _	**Finish Time**	_ _:_ _	
	**Hearing**			**Comments** (Insert Code)
**Do you use any of the following aids or appliances to help with your hearing?**	**Hearing aid all of the time**		**Y/N**	
	**Hearing aid some of the time**		**Y/N**	
	**Amplifier**		**Y/N**	
	**None of above**		**Y/N**	
	**Don't know**		**Y/N**	
	**Refused**		**Y/N**	
**Is your hearing (with or without a hearing aid)?**	**Excellent**		**Y/N**	
	**Very good**		**Y/N**	
	**Good**		**Y/N**	
	**Fair**		**Y/N**	
	**Poor**		**Y/N**	
	**Don't know**		**Y/N**	
	**Refused**		**Y/N**	
**Can you follow a conversation with one person (with or without a hearing aid)? If participant asks, clarify that the environment to think of should be non-noisy, i.e. their home**	**With no difficulty**		**Y/N**	
	**With some difficulty**		**Y/N**	
	**With much difficulty**		**Y/N**	
	**No I cannot**		**Y/N**	
	**Don't know**		**Y/N**	
	**Refused**		**Y/N**	
**Can you follow a conversation with four people (with or without a hearing aid)? If participant asks, clarify that the environment to think of should be non-noisy, i.e. their home**	**With no difficulty**		**Y/N**	
	**With some difficulty**		**Y/N**	
	**With much difficulty**		**Y/N**	
	**No I cannot**		**Y/N**	
	**Don't know**		**Y/N**	
	**Refused**		**Y/N**	
**Can you use a normal telephone? (A 'normal telephone' means a phone that has not been adapted for hearing impairment)**	**With no difficulty**		**Y/N**	
	**With some difficulty**		**Y/N**	
	**With much difficulty**		**Y/N**	
	**No I cannot**		**Y/N**	
	**Don't know**		**Y/N**	
	**Refused**		**Y/N**	

COMMENTS			
1	Refused Test	4	Test Incomplete
2	Physically Unable	5	Test Abandoned
3	Best Attempt	6	Other (please State)

NICOLA ID: | | | | | | |

Do you get or have you had noises (such as ringing or buzzing) in your head or in one or both ears that lasts for more than five minutes at a time?	Yes, now a lot of the time	Y/N	
	Yes, now most or all of the time	Y/N	
	Yes, now some of the time	Y/N	
	Yes, but not now, but have in the past	Y/N	
	No, never	Y/N	
	Don't know	Y/N	
	Refused	Y/N	
How much do these noises worry, annoy or upset you when they are at their worst?	Severely	Y/N	
	Moderately	Y/N	
	Slightly	Y/N	
	Not at all	Y/N	
	Don't know	Y/N	
	Refused	Y/N	
Have you ever worked in a noisy place where you had to shout to be heard	More than 5 years	Y/N	
	Less than 5 years	Y/N	
	Less than year	Y/N	
	Never	Y/N	
	Don't know	Y/N	
	Refused	Y/N	
Have you ever listened to music for more than 3 hours per week at a volume which you would need to shout to be heard or, if wearing headphones, someone else would need to shout for you to hear them?	More than 5 years	Y/N	
	Less than 5 years	Y/N	
	Less than year	Y/N	
	Never	Y/N	
	Don't know	Y/N	
	Refused	Y/N	

Recorded By:	Signature:	Date: _ _ / _ _ _ / _ _ _ _

COMMENTS			
1	Refused Test	4	Test Incomplete
2	Physically Unable	5	Test Abandoned
3	Best Attempt	6	Other (please State)

NICOLA ID: | | | | | | |

Vision			Comments (Insert Code)
Do you usually wear glasses or contact lenses? i.e. usually means most of time		Y/N	
Do you usually wear (choose the item worn most often)	Ordinary glasses	Y/N	
	Bifocals	Y/N	
	Contact lenses	Y/N	
	Varifocals	Y/N	
	Don't know	Y/N	
	Refused	Y/N	
How long have you had bifocals	Less than 1 year	Y/N	
	More than 1 year	Y/N	
	N/A	Y/N	
	Don't know	Y/N	
	Refused	Y/N	
Is your eyesight (using glasses or contact lens if you use them)	Excellent	Y/N	
	Very good	Y/N	
	Good	Y/N	
	Fair	Y/N	
	Poor	Y/N	
	Registered or legally blind	Y/N	
	Don't know	Y/N	
	Refused	Y/N	
How good is your eyesight for seeing things at a distance, like recognising a friend across the street (using glasses or contact lens if you use them)	Excellent	Y/N	
	Very good	Y/N	
	Good	Y/N	
	Fair	Y/N	
	Poor	Y/N	
	Don't know	Y/N	
	Refused	Y/N	

COMMENTS			
1	Refused Test	4	Test Incomplete
2	Physically Unable	5	Test Abandoned
3	Best Attempt	6	Other (please State)

NICOLA ID: | | | | | | |

How good is your eyesight for seeing things up close, like reading ordinary newspaper print (using glasses or contact lens if you use them)	Excellent	Y/N	
	Very good	Y/N	
	Good	Y/N	
	Fair	Y/N	
	Poor	Y/N	
	Don't know	Y/N	
	Refused	Y/N	
Has a doctor ever told you that you have any of the following eye diseases?	Cataracts	Y/N	
	Glaucoma	Y/N	
	Age Related Macular Degeneration	Y/N	
	Diabetic Retinopathy	Y/N	
	Other: please state:	Y/N	
Have you had cataract surgery	Rt. eye	Y/N	
	Lt. eye	Y/N	
	Never	Y/N	
	Don't know	Y/N	
	Refused	Y/N	
In the last 12 months, how often did you visit your optician?	__ days ago<br>_ _ weeks ago<br>_ _ months ago		
About how much did you pay out- of-pocket for visiting opticians, buying glasses or contact lenses in the last 12 months?(Amount of money on top of benefit which would cover some costs)	£_ _ _ _ : _ _		

Recorded By:	Signature:	Date: _ _ / _ _ _ / _ _ _ _

COMMENTS			
1	Refused Test	4	Test Incomplete
2	Physically Unable	5	Test Abandoned
3	Best Attempt	6	Other (please State)

NICOLA ID: | | | | | | |

Visual Acuity			Comments (Insert Code)
	RIGHT EYE	LEFT EYE	
Distance glasses worn for test	Y/N	Y/N	
Corrective lens worn for test	Y/N	Y/N	

IOP					Comments (Insert Code)
No IOP measurements to be taken if eye surgery in last 4 weeks					
IOP	Results	Rt Eye	Results	Lt Eye	
	IOPg1		IOPg1		
	IOPg2		IOPg2		
	IOPg3		IOPg3		
	IOPg Average		IOPg Average		

AutoRefractor		Comments (Insert Code)
Printout	Y/N	

Eye Drops					Comments (Insert Code)
Do not Dilate or undertake Retinal Imaging if Participant has Photosensitive Epilepsy					
Eye Drops	Right Eye	Y/N	Left Eye	Y/N	Comments (Insert Code)
Time: _ _ : _ _					
Administered By:					

Recorded By:	Signature:	Date: _ _ / _ _ _ / _ _ _ _

COMMENTS			
1	Refused Test	4	Test Incomplete
2	Physically Unable	5	Test Abandoned
3	Best Attempt	6	Other (please State)

NICOLA ID: | | | | | | |

1							NF	P/H	NF	P/H						
**M**	C	O	H	Z	V	5					5	Z	R	K	D	C
**E**	S	Z	N	D	C	10					10	D	N	C	H	V
**T**	V	K	C	N	R	15					15	C	D	H	N	R
**R**	K	C	R	H	N	20					20	R	V	Z	O	S
**E**	Z	K	D	V	C	25					25	O	S	D	V	Z
	H	V	O	R	K	30					30	N	O	Z	C	D
**4**	C	O	H	Z	V	35					35	Z	R	K	D	C
**M**	S	Z	N	D	C	40					40	D	N	C	H	V
**E**	V	K	C	N	R	45					45	C	D	H	N	R
**T**	K	C	R	H	N	50					50	R	V	Z	O	S
**R**	Z	K	D	V	C	55					55	O	S	D	V	Z
**E**	H	V	O	R	K	60					60	N	O	Z	C	D
	R	H	S	O	N	65					65	R	D	N	S	K
	K	S	V	R	H	70					70	O	K	S	V	Z
	H	N	K	C	D	75					75	K	S	N	H	O
	N	D	V	K	O	80					80	H	O	V	S	N
	D	H	O	S	Z	85					85	V	C	S	Z	H
	V	R	N	D	O	90					90	C	Z	D	R	V
	C	Z	H	K	S	95					95	S	H	R	Z	C
	O	R	Z	S	K	100					100	D	N	O	K	R
Total :																

RE (tick)	COMMENTS:	LE (tick)
CF		CF
HM		HM
LP		LP
NO LP		NO LP

Recorded By:	Signature:	Date: _ _ / _ _ _ / _ _ _ _

COMMENTS			
1	Refused Test	4	Test Incomplete
2	Physically Unable	5	Test Abandoned
3	Best Attempt	6	Other (please State)

NICOLA ID: | | | | | | |

**Anthropometric**				**Comments** (Insert Code)
	1st	2nd	3rd if req.	
**Waist** (nearest mm)	_ _ _ . _	_ _ _ . _	_ _ _ . _	
**Hip** (nearest mm)	_ _ _ . _	_ _ _ . _	_ _ _ . _	
**Height (nearest cm)**	_ _ _ **cm**	**Weight (to 1 decimal place)**	_ _ _ . _ **kg**	

**Cardiovascular**					**Comments** (Insert Code)
**Blood Pressure**	Systolic	Diastolic	Pulse	Room Temp _ _: _ ⁰C	
**BP 1**	_ _ _	_ _ _	_ _ _		
**BP 2**	_ _ _	_ _ _	_ _ _		
**BP 3**	_ _ _	_ _ _	_ _ _		
**On Standing: Circle Response**	Dizzy **Y/N**	Lightheaded **Y/N**		Unsteady **Y/N**	
**BP Interpretation: Circle Interpretation**	Low	Normal		Elevated	
**Nurse Action/ Comments**					

**Gait & Balance**				**Comments** (Insert Code)
**Step Test**	Dominant Side Right / Left	N0 of Steps		
		Right foot<br>_ _	Left foot<br>_ _	
**Time Up & Go** (to 2 dec places)	| | | :	|		

**Recorded By:**	**Signature:**	**Date:** _ _ / _ _ _ / _ _ _ _

COMMENTS			
1	Refused Test	4	Test Incomplete
2	Physically Unable	5	Test Abandoned
3	Best Attempt	6	Other (please State)

NICOLA ID: | | | | | | |

**Grip Strength**				**Comments** (Insert Code)
(nearest whole number)	Non-Dom. Hand	Right/Left	_ _ KG	
	Dominant Hand	Right/Left	_ _ KG	
	Non-Dom. Hand	Right/Left	_ _ KG	
	Dominant Hand	Right/Left	_ _ KG	

**Bloods**		**Comments** (Insert Code)
Both Arms Suitable for Venepuncture	**Y/N**	
Have you been diagnosed with a clotting or bleeding disorder?	**Y/N**	
Time of last meal	_ _ : _ _	
Venepuncture Time	_ _ : _ _	
Yellow Top 4	**Y/N**	
PAXgene RNA 1	**Y/N**	
Purple Top 4	**Y/N**	
Grey Top 1	**Y/N**	

**Other Tests**			**Comments** (Insert Code)
**Bodystat**	**Printout**	**Y/N**	
**Urine**		**Y/N**	
**Photograph**		**Y/N**	
**Spirometry**		**Y/N**	

**Recorded By:**	**Signature:**	**Date:** _ _ / _ _ _ / _ _ _ _

COMMENTS			
1	Refused Test	4	Test Incomplete
2	Physically Unable	5	Test Abandoned
3	Best Attempt	6	Other (please State)

NICOLA ID: | | | | | | |

**Vision**					**Comments** (Insert Code)
	**Rt Eye**		**Lt Eye**		
**Canon CX-1**	**Fundus Reflex**	**Y/N**	**Fundus Reflex**	**Y/N**	
	**Field 1M (Optic)**	**Y/N**	**Field 1M (Optic)**	**Y/N**	
	**Field 2M (Macula)**	**Y/N**	**Field 2M (Macula)**	**Y/N**	
	**Stereoscopic Optic Disc**	**Y/N**	**Stereoscopic Optic Disc**	**Y/N**	
	**Stereoscopic Macula**	**Y/N**	**Stereoscopic Macula**	**Y/N**	
**OPTOS**	**Colour Optomap Plus Images 2 on Axes**	**Y/N**	**Colour Optomap Plus Images 2 on Axes**	**Y/N**	
	**AF Optomap Plus 1 on Axes**	**Y/N**	**AF Optomap Plus 1 on Axes**	**Y/N**	
**Spectralis**	**P Pole**	**Y/N**	**P Pole**	**Y/N**	
	**RNFL**	**Y/N**	**RNFL**	**Y/N**	
	**Line EDI**	**Y/N**	**Line EDI**	**Y/N**	
	**Multi**	**Y/N**	**Multi**	**Y/N**	
	**qAF**	**Y/N**	**qAF**	**Y/N**	
	**Blue AF Movie**	**Y/N**	**Blue AF Movie**	**Y/N**	

**Recorded By:**	**Signature:**	**Date:** _ _ / _ _ _ / _ _ _ _

COMMENTS			
1	Refused Test	4	Test Incomplete
2	Physically Unable	5	Test Abandoned
3	Best Attempt	6	Other (please State)

NICOLA ID: | | | | | | |

MINI MENTAL STATE EXAMINATION (MMSE)

**COMMENTS:**		
**Recorded By:**	**Signature:**	**Date:** _ _ / _ _ _ / _ _ _ _

COMMENTS			
1	Refused Test	4	Test Incomplete
2	Physically Unable	5	Test Abandoned
3	Best Attempt	6	Other (please State)

NICOLA ID: | | | | | | |

Colour Trails 2

**Time** : _ _: _ _: _ _	**Total Score:**
**Colour Errors :**	_ _
**Number Errors :**	_ _
**Prompts :**	_ _
**Near misses :**	_ _

**COMMENTS:**		
**Recorded By:**	**Signature:**	**Date:** _ _ / _ _ _ / _ _ _ _

COMMENTS			
1	Refused Test	4	Test Incomplete
2	Physically Unable	5	Test Abandoned
3	Best Attempt	6	Other (please State)

NICOLA ID: | | | | | | |

MoCA Test:

Animal Recall:

Score:

_ _

COMMENTS:

COMMENTS			
1	Refused Test	4	Test Incomplete
2	Physically Unable	5	Test Abandoned
3	Best Attempt	6	Other (please State)

NICOLA ID: | | | | | | |

**MOCA and Animal Recall Recorded By:**	**Signature:**	**Date: _ _ / _ _ _ / _ _ _ _**

CES-D

Circle Answer Number

1) I was bothered by things that usually don't bother me

'WOULD YOU SAY THIS STATEMENT DESCRIBES THE WAY YOU FELT DURING THE PAST WEEK

1. Rarely or none of the time (less than 1 day).
2. Some or little of the time (1-2 days).
3. Occasionally or a moderate amount of time (3-4 days).
4. All of the time (5-7 days).
5. Don't Know
6. Refused

2) I did not feel like eating; my appetite was poor.

'WOULD YOU SAY THIS STATEMENT DESCRIBES THE WAY YOU FELT DURING THE PAST WEEK

1. Rarely or none of the time (less than 1 day).
2. Some or little of the time (1-2 days).
3. Occasionally or a moderate amount of time (3-4 days).
4. All of the time (5-7 days).
5. Don't Know
6. Refused

3) I felt that I could not shake off the blues even with help from my family or friends.

'WOULD YOU SAY THIS STATEMENT DESCRIBES THE WAY YOU FELT DURING THE PAST WEEK

1. Rarely or none of the time (less than 1 day).
2. Some or little of the time (1-2 days).
3. Occasionally or a moderate amount of time (3-4 days).
4. All of the time (5-7 days).
5. Don't Know
6. Refused

4) I felt that I was just as good as other people.

'WOULD YOU SAY THIS STATEMENT DESCRIBES THE WAY YOU FELT DURING THE PAST WEEK

1. Rarely or none of the time (less than 1 day).
2. Some or little of the time (1-2 days).
3. Occasionally or a moderate amount of time (3-4 days).
4. All of the time (5-7 days).
5. Don't Know
6. Refused

5) I had trouble keeping my mind on what I was doing.

'WOULD YOU SAY THIS STATEMENT DESCRIBES THE WAY YOU FELT DURING THE PAST WEEK

1. Rarely or none of the time (less than 1 day).
2. Some or little of the time (1-2 days).
3. Occasionally or a moderate amount of time (3-4 days).
4. All of the time (5-7 days).
5. Don't Know
6. Refused

COMMENTS			
1	Refused Test	4	Test Incomplete
2	Physically Unable	5	Test Abandoned
3	Best Attempt	6	Other (please State)

NICOLA ID:

**Comments:**		
**Recorded By:**	**Signature:**	**Date:** _ _ / _ _ _ / _ _ _ _

6) I felt depressed.
'WOULD YOU SAY THIS STATEMENT DESCRIBES THE WAY YOU FELT DURING THE PAST WEEK

1. Rarely or none of the time (less than 1 day).
2. Some or little of the time (1-2 days).
3. Occasionally or a moderate amount of time (3-4 days).
4. All of the time (5-7 days).
5. Don't Know
6. Refused

7) I felt that everything I did was an effort.
'WOULD YOU SAY THIS STATEMENT DESCRIBES THE WAY YOU FELT DURING THE PAST WEEK

1. Rarely or none of the time (less than 1 day).
2. Some or little of the time (1-2 days).
3. Occasionally or a moderate amount of time (3-4 days).
4. All of the time (5-7 days).
5. Don't Know
6. Refused

8) I felt hopeful about the future.
'WOULD YOU SAY THIS STATEMENT DESCRIBES THE WAY YOU FELT DURING THE PAST WEEK

1. Rarely or none of the time (less than 1 day).
2. Some or little of the time (1-2 days).
3. Occasionally or a moderate amount of time (3-4 days).
4. All of the time (5-7 days).
5. Don't Know
6. Refused

9) I thought my life had been a failure.
'WOULD YOU SAY THIS STATEMENT DESCRIBES THE WAY YOU FELT DURING THE PAST WEEK

1. Rarely or none of the time (less than 1 day).
2. Some or little of the time (1-2 days).
3. Occasionally or a moderate amount of time (3-4 days).
4. All of the time (5-7 days).
5. Don't Know
6. Refused

10) I felt fearful.
'WOULD YOU SAY THIS STATEMENT DESCRIBES THE WAY YOU FELT DURING THE PAST WEEK

1. Rarely or none of the time (less than 1 day).
2. Some or little of the time (1-2 days).
3. Occasionally or a moderate amount of time (3-4 days).
4. All of the time (5-7 days).
5. Don't Know
6. Refused

COMMENTS			
1	Refused Test	4	Test Incomplete
2	Physically Unable	5	Test Abandoned
3	Best Attempt	6	Other (please State)

NICOLA ID: | | | | | |

Comments:		
Recorded By:	Signature:	Date: _ _ / _ _ _ / _ _ _ _

11) My sleep was restless.
'WOULD YOU SAY THIS STATEMENT DESCRIBES THE WAY YOU FELT DURING THE PAST WEEK

1. Rarely or none of the time (less than 1 day).
2. Some or little of the time (1-2 days).
3. Occasionally or a moderate amount of time (3-4 days).
4. All of the time (5-7 days).
5. Don't Know
6. Refused

12) I was happy.
'WOULD YOU SAY THIS STATEMENT DESCRIBES THE WAY YOU FELT DURING THE PAST WEEK

1. Rarely or none of the time (less than 1 day).
2. Some or little of the time (1-2 days).
3. Occasionally or a moderate amount of time (3-4 days).
4. All of the time (5-7 days).
5. Don't Know
6. Refused

13) I talked less than usual.
'WOULD YOU SAY THIS STATEMENT DESCRIBES THE WAY YOU FELT DURING THE PAST WEEK

1. Rarely or none of the time (less than 1 day).
2. Some or little of the time (1-2 days).
3. Occasionally or a moderate amount of time (3-4 days).
4. All of the time (5-7 days).
5. Don't Know
6. Refused

14) I felt lonely.
'WOULD YOU SAY THIS STATEMENT DESCRIBES THE WAY YOU FELT DURING THE PAST WEEK

1. Rarely or none of the time (less than 1 day).
2. Some or little of the time (1-2 days).
3. Occasionally or a moderate amount of time (3-4 days).
4. All of the time (5-7 days).
5. Don't Know
6. Refused

15) People were unfriendly.
'WOULD YOU SAY THIS STATEMENT DESCRIBES THE WAY YOU FELT DURING THE PAST WEEK

1. Rarely or none of the time (less than 1 day).
2. Some or little of the time (1-2 days).
3. Occasionally or a moderate amount of time (3-4 days).
4. All of the time (5-7 days).
5. Don't Know
6. Refused

COMMENTS			
1	Refused Test	4	Test Incomplete
2	Physically Unable	5	Test Abandoned
3	Best Attempt	6	Other (please State)

NICOLA ID: | | | | | |

Comments:		
Recorded By:	Signature:	Date: _ _ / _ _ _ / _ _ _ _

16) I enjoyed life.
'WOULD YOU SAY THIS STATEMENT DESCRIBES THE WAY YOU FELT DURING THE PAST WEEK
1. Rarely or none of the time (less than 1 day).
2. Some or little of the time (1-2 days).
3. Occasionally or a moderate amount of time (3-4 days).
4. All of the time (5-7 days).
5. Don't Know
6. Refused

17) I had crying spells.
'WOULD YOU SAY THIS STATEMENT DESCRIBES THE WAY YOU FELT DURING THE PAST WEEK
1. Rarely or none of the time (less than 1 day).
2. Some or little of the time (1-2 days).
3. Occasionally or a moderate amount of time (3-4 days).
4. All of the time (5-7 days).
5. Don't Know
6. Refused

18) I felt sad.
'WOULD YOU SAY THIS STATEMENT DESCRIBES THE WAY YOU FELT DURING THE PAST WEEK
1. Rarely or none of the time (less than 1 day).
2. Some or little of the time (1-2 days).
3. Occasionally or a moderate amount of time (3-4 days).
4. All of the time (5-7 days).
5. Don't Know
6. Refused

19) I felt that people disliked me.
'WOULD YOU SAY THIS STATEMENT DESCRIBES THE WAY YOU FELT DURING THE PAST WEEK
1. Rarely or none of the time (less than 1 day).
2. Some or little of the time (1-2 days).
3. Occasionally or a moderate amount of time (3-4 days).
4. All of the time (5-7 days).
5. Don't Know
6. Refused

20) I could not get "going."
'WOULD YOU SAY THIS STATEMENT DESCRIBES THE WAY YOU FELT DURING THE PAST WEEK
1. Rarely or none of the time (less than 1 day).
2. Some or little of the time (1-2 days).
3. Occasionally or a moderate amount of time (3-4 days).
4. All of the time (5-7 days).
5. Don't Know
6. Refused

COMMENTS			
1	Refused Test	4	Test Incomplete
2	Physically Unable	5	Test Abandoned
3	Best Attempt	6	Other (please State)

NICOLA ID: | | | | | | |

**Comments:**		
**Recorded By:**	**Signature:**	**Date: _ _ / _ _ _ / _ _ _ _**

COMMENTS			
1	Refused Test	4	Test Incomplete
2	Physically Unable	5	Test Abandoned
3	Best Attempt	6	Other (please State)
